# Surgical approximation of the posterior papillary muscle in chronic ischemic mitral regurgitation – presentation of a new method of mitral valve repair

**DOI:** 10.1186/1749-8090-8-S1-O268

**Published:** 2013-09-11

**Authors:** N Alotti, K Gombocz, A Rashed, Cs Dézsi, A Sayur

**Affiliations:** 1 Cardiac Surgery Department of Zala County Hospital & Pécs University, Zalaegerszeg, Hungary; 2 Cardiology Department of Petz Aladár Hospital, Győr, Hungary

## Background

The optimal surgical management of the chronic ischemic mitral regurgitation remains controversial and the negative effect of the displacement of the papillary muscles is evident. To emerge this effect we performed a new method to approximate the papillary muscles as adjunct procedure to ring annuloplasty. The purpose of this study was to evaluate the early outcome and the effect of this method.

## Methods

Between 2007 and 2012 108 patients (mean age 63.8 years; range: 48-78 years) with a preoperative diagnosis of functional MR underwent either annuloplasty ± new chordal implantation (group AC, N=56) or annuloplasty + papillary muscle approximation + new chordal implantation (group AAC, N=52) for mitral valve repair along with myocardial revascularization (1.9 ± 1.0 distal anastomoses per patients). A Carpentier ring was always used in the repair procedures. Papillary muscle approximation was achieved with auto-pericardium pledgeted U shaped, Bridge like polytetrafluoroethylene suture placed between the head of the two papillary muscles, creating a distance ≤ 15 mm. We compared echocardiographic and clinical data for these two groups. The two groups were homogeneous for preoperative characteristics.

## Results

Follow-up was 98.2% and 100% complete in the AC and AAC groups, respectively. No operative mortality was observed. At 5 years, overall survival was 89,3 in the AC group, and 94,2% in the AAC group (P = .05) No reoperation was required. 6 months after the operation 91 patients showed no leakage, 9 had trivial regurgitation and 8 had regurgitation grade ≥ 1 (44, 5, 7 in AC group, 47, 4, 1 in AAC group respectively) New York Heart Association functional class I or II was documented in 92.3% of patients in the AC group and 95.9% in the AAC group (P = .1).

## Conclusions

Papillary muscle approximation as adjunct procedure to annuloplasty in functional MR was valuable for eliminating MR. However, this technique may influence long term outcome and survival.

